# The Early Phase of β2-Microglobulin Aggregation: Perspectives From Molecular Simulations

**DOI:** 10.3389/fmolb.2020.578433

**Published:** 2020-09-29

**Authors:** Rui J. S. Loureiro, Patrícia F. N. Faísca

**Affiliations:** ^1^Faculty of Sciences, BioISI – Biosystems & Integrative Sciences Institute, University of Lisboa, Lisbon, Portugal; ^2^Department of Physics, Faculty of Sciences, University of Lisboa, Lisbon, Portugal

**Keywords:** protein aggregation, molecular dynamics, docking, dimer, intermediates, native-centric simulations

## Abstract

Protein β2-microglobulin is the causing agent of two amyloidosis, dialysis related amyloidosis (DRA), affecting the bones and cartilages of individuals with chronic renal failure undergoing long-term hemodialysis, and a systemic amyloidosis, found in one French family, which impairs visceral organs. The protein’s small size and its biomedical significance attracted the attention of theoretical scientists, and there are now several studies addressing its aggregation mechanism in the context of molecular simulations. Here, we review the early phase of β2-microglobulin aggregation, by focusing on the identification and structural characterization of monomers with the ability to trigger aggregation, and initial small oligomers (dimers, tetramers, hexamers etc.) formed in the so-called nucleation phase. We focus our analysis on results from molecular simulations and integrate our views with those coming from *in vitro* experiments to provide a broader perspective of this interesting field of research. We also outline directions for future computer simulation studies.

## Introduction

Protein folding is the self-assembly process according to which a linear polypeptide chain acquires a soluble, three-dimensional structure that is biologically functional. Protein aggregation is the self-association process in which soluble protein conformations (monomers) interact with each other forming dimers, trimers, tetramers and higher order oligomers (Gomes and Faísca, 2019). Generally, protein aggregation leads to amorphous aggregates with a granular appearance, protofibrils (including annular oligomeric aggregates), and other oligomeric aggregated states. Sometimes, however, the end product of protein aggregation are the so-called amyloids, insoluble aggregates comprising long unbranched fibers, characterized by the cross-beta structure, i.e., an extended beta-sheet secondary structure in which individual monomers arranged as β-strands are stacked strictly above each other perpendicularly to the fiber’s axis (Eisenberg and Jucker, 2012).

Amyloid structure may have a functional role (Otzen and Riek, 2019), but it is often associated with disease. So far, over 50 diseases have been linked with amyloid (Chiti and Dobson, 2006). Examples of the so-called conformational disorders include the well-known Alzheimer’s disease, Parkinson’s disease, cataracts, type II diabetes, and some forms of cancer. A less familiar pathology is dialysis related amyloidosis (DRA) (Gejyo et al., 1985; Kiss et al., 2005; Corlin, 2012), affecting individuals with chronic renal failure undergoing long-term (> 10 years) hemodialysis (Floege and Ketteler, 2001; Esposito et al., 2012). The pathological process of DRA is underpinned by a 60-fold increase of the plasmatic concentration of protein β2-microglobulin (β2m) (Floege and Ketteler, 2001; Eichner and Radford, 2011), which results from the incapacity of the kidney to catabolize the protein and failure of the dialysis apparatus to filter it. The protein’s high affinity for collagen and its deposition in bones and cartilages (Giorgetti et al., 2005; Esposito et al., 2012)—where it eventually oligomerizes into amyloids—causes destructive arthropathy, cystic bone lesions, carpal tunnel syndrome (and other neuropathies), joint pain, impaired function, and bone fractures.

Classically, protein folding and protein aggregation are viewed as independent processes, with the latter being initiated by some drastic conformational change of the native structure triggered by harsh environmental conditions (low pH, high temperature, presence of chemical denaturants, etc.), or induced by some severely destabilizing mutation (Kelly, 1998). However, evidence accumulated in the last two decades revealed a different picture of protein aggregation. According to the current view, aggregation can actually be triggered by an unfolded nativelike state, which becomes populated as a result of thermal fluctuations occurring under physiological conditions, or by an intermediate (mis)folding state competent to form amyloid (Thirumalai et al., 2003; Chiti and Dobson, 2009). The identification of monomeric species that can fold to the native state and also initiate the amyloid cascade, prompted a new view of protein aggregation and highlighted a much direct connection (in some cases a direct competition) between the two processes (Bratko and Blanch, 2001; Jahn and Radford, 2008). β2m is a paradigmatic model system for studying the connection between the two processes since it populates an intermediate state that can either fold to the native structure, or trigger the amyloid cascade (Jahn et al., 2006).

**FIGURE 1:**
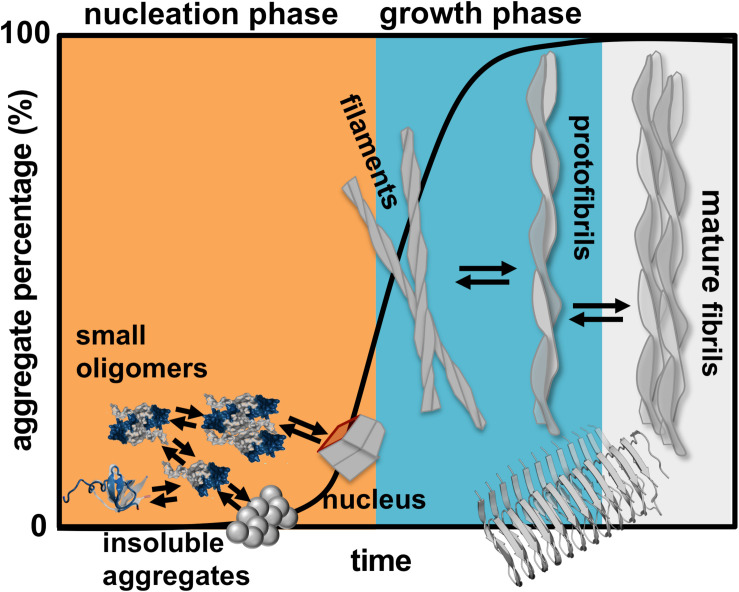
Schematics of protein aggregation. Representation of the process of protein aggregation as a function of time, starting from aggregation prone monomers till the formation of mature amyloid fibrils. This article focus on the nucleation phase, whereby small oligomers (dimers, trimers, tetramers etc.) form prior to the establishment of an aggregation nucleus that triggers the growth phase leading to fibrils.

Finding a cure for DRA, and for amyloid disease in general, requires the determination of the aggregation mechanism leading to amyloids starting from aggregation prone monomers. Protein aggregation involves the formation of molecular structures with up to hundreds of nanometers, and timescales that can extend to hours (Figure 1). In rigor, establishing the mechanism of aggregation implies the identification of all microscopic steps leading to mature fibrils, the rate constants governing each step (i.e., the kinetics), and determining the manner according to which they depend on protein sequence and environmental conditions. Ideally, it should be possible to determine the size distribution and structures of the oligomeric assemblies, filaments, protofibrils and fibrils that populate the amyloid pathway (Cohen et al., 2012). In particular, the structural characterization of the first steps of oligomer formation, including the identification of monomeric species capable of self-association, is key for understanding the aggregation process, and critical for the design of therapeutic drugs that have the ability to block it.

On the experimental side, X-ray diffraction (X-ray), nuclear magnetic resonance (NMR)—both in solution and in the solid state, mass spectrometry (MS), and, more recently, cryo-electron microscopy (cryo-EM) have all been extensively used to structurally characterize monomers (Eichner et al., 2011; Le Marchand et al., 2018), oligomers (Mendoza et al., 2010, 2011), and amyloid fibrils (White et al., 2009; Iadanza et al., 2018). The structure and morphology of amyloids provides clues onto the aggregation mechanism of several proteins, including that of β2m (White et al., 2009; Iadanza et al., 2018). However, the structural characterization with atomic resolution of aggregation-prone monomeric species and early-formed oligomers is particularly challenging (Wei et al., 2007). Indeed, these species exist in a complex dynamic equilibrium with each other, and with insoluble higher order aggregates. Furthermore, they form transiently (i.e., in a timescale that is not compatible with the temporal resolution of commonly used biophysics apparatus), and in a manner that is strongly dependent on environmental conditions.

In principle, molecular simulations should allow isolating every conformational state populated along the amyloid pathway with the desired temporal resolution. They stand, therefore, as a potentially important methodology to study protein aggregation from a structural and dynamical standpoint. In particular, by using of single or combined computational methodologies one should be able to cover larger time-scales and predict aggregation-prone monomers and early-formed oligomers (dimers, trimers, tetramers etc.). The structural characterization of the latter allows determining the most likely adhesion zones and, at an atomic level, predicting the residues that play a critical role in stabilizing the oligomer’s interface. These theoretical predictions can be used to interpret existing experimental data and guide novel *in vitro* experiments, thus complementing experimental studies.

Here, we review a collection of results (including our own work) obtained in the framework of molecular simulations that shed light onto the mechanism of β2m aggregation. Concretely, our focus is the formation and structural characterization of the initial species that populate the aggregation pathway, including monomers, dimers and other small oligomers. In kinetic terms these species comprise the so-called nucleation phase, i.e., the phase that terminates with the formation of a critical nucleus that has the ability to grow into a small fibrillar structure (Figure 1).

In what follows we present an outline of the computational methods and physical models currently used to study protein aggregation. Subsequently, we provide an overview of the specific model systems that have been deployed to study β2m aggregation *in vitro* and in the computer. In the core part of this review, we blend findings from molecular simulations with experimental results to provide a broader view of the early phase regulating this remarkable complex phenomenon. We finally draw some conclusions and outline some questions for future research.

## Computational Models and Techniques Used to Study β2m Aggregation

In what follows we provide a brief summary of current state-of-the-art methodologies used to explore protein aggregation in the scope of molecular simulations. The interested reader should refer to recent reviews for a more comprehensive view of this matter (Ma and Nussinov, 2006; Wei et al., 2007; Redler et al., 2014; Morriss-Andrews and Shea, 2015).

Since the timescale of protein aggregation is exceedingly large, it is not possible to use classical Molecular Dynamics (MD) of all atom (AA) models to follow and monitor the whole process. Indeed, AA models represent both protein and water with atomistic detail, and this fine-grained representation comes at a high computational cost that strongly limits the accessible timescales. To the best of our knowledge the longest MD simulation carried out so far lasted ∼1 ms in the Anton supercomputer (Lindorff-Larsen et al., 2011), with commercial machines typically covering the microsecond timescale. Therefore, MD simulations of AA models are used to primarily study monomers and very small oligomers, as well as the stability of pre-formed amyloid fibrils, and their interaction with other molecules (Morriss-Andrews and Shea, 2015).

A popular workaround to overcome the limitations of classical MD is the replica exchange (RE) method (Nishino et al., 2005; Liang et al., 2007, 2008; Wei et al., 2008; De Simone and Derreumaux, 2010; Nishikawa et al., 2019), in which several MD trajectories (the so called replicas) run in parallel at different temperatures, and moves between adjacent replicas are attempted based on a Monte Carlo Metropolis rule (Nishino et al., 2005; Liang et al., 2007, 2008; Wei et al., 2008; De Simone and Derreumaux, 2010; Nishikawa et al., 2019). By limiting trapping in local energy minima, RE allows equilibrium sampling of the conformational landscape. However, since the constant temperature trajectories are discontinuous, the RE sampling method loses kinetic information. Furthermore, since the number of replicas increases markedly with system’s size, the use of RE-MD is limited to the study of oligomerization of small peptide fragments (Nishino et al., 2005; Liang et al., 2007, 2008; Wei et al., 2007; De Simone and Derreumaux, 2010; Nishikawa et al., 2019). In the case of β2m, MD simulations have been used to study the dynamics of the native monomer (Ma and Nussinov, 2003; Deng et al., 2006; Fogolari et al., 2011; Chandrasekaran and Rajasekaran, 2016), the oligomerization process of selected amyloidogenic β2m peptide sequences (Liang et al., 2007; Zheng et al., 2008; Fang et al., 2009a), and provide complementary information to that obtained through *in vitro* experiments (Gumral et al., 2013; Chong et al., 2015; Torbeev et al., 2015; Camilloni et al., 2016; Hall et al., 2016; Brancolini et al., 2018). Another enhanced sampling scheme is the so-called metadynamics (Laio and Parrinello, 2002). In this case sampling is facilitated by an additional bias potential (or force) acting on a selected number of degrees of freedom, often referred to as collective variables (Barducci et al., 2011). Recently, a method that incorporates experimental data as replica-averaged structural restraints in MD simulations, based on the metadynamics framework (Camilloni et al., 2013) has been applied to study β2m (Le Marchand et al., 2018). Another way to speed up conformational sampling is to represent the solvent implicitly such as in the Generalized Born model (Lei et al., 2006). Implicit solvent MD simulations have been used to study the dimerization phase of β2m aggregation (Lei et al., 2006).

In order to access long timescales, associated with large conformational changes it is necessary to use coarse-grained (CG) models. These models consider a reduced representation of the protein, in which a selected group of atoms is represented by a single bead (generally termed super atom) following a suitable mapping scheme. The simplest CG model is a lattice model in which each amino acid is reduced to a bead located on the vertices of a regular lattice embedded in the 2D or 3D space. Lattice models have a long history in the study of protein folding (Sali et al., 1994; Mirny et al., 1996; Pande and Rokhsar, 1999; Faisca et al., 2004, 2010), and they have also been used to investigate protein aggregation (Li et al., 2008; Abeln et al., 2014). Additionally, several CG models of intermediate resolution have been developed such as the UNRES (Czaplewski et al., 2018), PRIME20 (Cheon et al., 2012), MARTINI (Monticelli et al., 2008), OPEP (Song et al., 2008; Wei et al., 2008; Lu et al., 2009; De Simone and Derreumaux, 2010; Sterpone et al., 2014), amongst others (Bellesia and Shea, 2007; Friedman and Caflisch, 2011; Carrillo-Parramon et al., 2016). The interaction potential used in CG models needs to be parameterized, either following a knowledge-based or a structure-based approach (Kmiecik et al., 2016). The simplest energy potential used in combination with a CG representation is the structure-based Gō potential (Taketomi et al., 1975), which only considers the contribution of native interactions to the protein’s energy, being, therefore, not transferable. Also, since simple Gō potentials do not incorporate non-native interactions, they will not be able to capture misfolding processes leading to compact non-native states that can trigger aggregation, or, more generally, regions of the folding free energy landscape where non-native interactions may play a determinant role (e.g., the denatured state). Symmetrized Gō potentials have been used to study the direct competition of protein folding and protein aggregation by considering that an intermolecular contact is stabilizing if the interacting pair of beads is also involved in a native contact at the monomer level (i.e., in an intramolecular interaction). Typically, these modified Gō potentials lead to domain-swapping (DS), a process of self-association in which two monomers exchange (one or more) identical parts of their structure (loops, helices or beta-strands) forming a bound dimer (Ding et al., 2002). It is also possible to combine a full atomistic representation of the protein with the use of square-well discontinuous interaction potentials, including the Gō potential (Estacio et al., 2012, 2013, 2014; Krobath et al., 2012; Loureiro et al., 2017, 2019), and sample the conformational space with discontinuous molecular dynamics (DMD) simulations (Chen and Dokholyan, 2005; Estacio et al., 2012, 2013, 2014; Krobath et al., 2012; Loureiro et al., 2017, 2019), an efficient, event driven sampling scheme, which can also be combined with RE. Faísca et al. have recently employed RE-DMD to compute the equilibrium folding space of the protein domain spcSH3 (Krobath et al., 2012) and β2m to predict aggregation prone folding intermediates (Estacio et al., 2014; Loureiro et al., 2017, 2019).

Another set of computational methodologies that can be used to obtain structural models of dimers and higher order oligomers is protein-protein docking. In particular, docking methods based on a rigid-body representation allow for efficient sampling of the conformational space (Norel et al., 1994, 1995, 1999; Benyamini et al., 2003, 2005; Kozakov et al., 2006; Krobath et al., 2012). The scoring function used in protein-protein docking can be based on shape complementarity only (Lawrence and Colman, 1993; Norel et al., 1994, 1995, 1999; Krobath et al., 2012), or consider the other main drivers of protein-protein association, which are hydrophobic and electrostatic interactions (Dominguez et al., 2003; Fernandez-Recio et al., 2003; Gray et al., 2003; Mendoza et al., 2010, 2011; Cantarutti et al., 2017; Brancolini et al., 2018; Loureiro et al., 2019). Some docking methodologies also include restraints derived from experiments (Dominguez et al., 2003; Karamanos et al., 2014; Hall et al., 2016); this is the case of HADDOCK, which is available as a webserver (Dominguez et al., 2003; de Vries et al., 2010).

**FIGURE 2:**
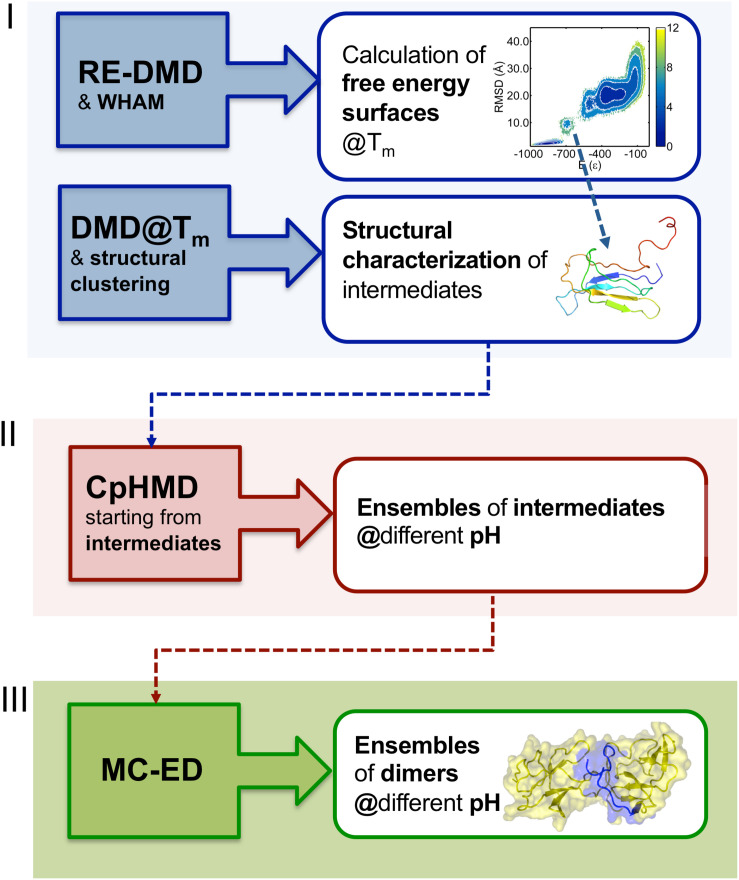
Three-stage methodology to investigate the early phase of protein aggregation. The conformational space is explored with RE-DMD of a full atomistic Gō model to compute equilibrium folding thermodynamics (including the melting temperature, Tm, and free energy surfaces) to detect the formation of intermediate states). Large ensembles of intermediates are collected from DMD simulations at constant temperature, and structurally characterized to select those with aggregation potential. Representative conformations are then subjected to a CpHMD protocol with explicit titration to obtain ensembles of intermediates representative of different pH conditions. The latter are finally used in a protein-protein docking protocol that creates ensembles of dimers whose statistical analysis allows determining the most likely regions and residues involved in the onset of aggregation.

In order to study the early phase of aggregation of β2m by means of molecular simulations, Faísca et al. developed a methodology that combines and integrates data obtained from several computational tools mentioned above, namely, RE-DMD simulations, constant pH Molecular Dynamics (CpHMD) simulations, and protein-protein docking simulations (Estacio et al., 2014; Loureiro et al., 2017, 2019). This methodology does not consider the possibility of protein dimerization occurring concurrently and concomitantly with folding—a situation that could lead to DS dimers—but considers instead the scenario according to which protein–protein association occurs upon the formation of aggregation prone intermediate states *en route* to the native state. Such folding intermediates must be thermodynamically stable enough (i.e., sufficiently long-lived) to allow for the establishment of intermolecular interactions. Accordingly, the methodology comprises three stages (Figure 2). RE-DMD simulations are used in the first stage, which consists in the exploration of the folding transition of a full atomistic Gō model to identify nativelike folding intermediate(s) with aggregation potential (e.g., by exposing hydrophobic patches). In the second stage, the folding intermediate(s) are used as starting conformations of CpHMD simulations (with explicit titration) (Baptista et al., 2002; Machuqueiro and Baptista, 2006). The latter allows accurately capturing the effects of pH on protein structure. Indeed, the pH modulates the charge of the ionizable side-chains, which can induce large-scale conformational changes (e.g., the modification of secondary/tertiary structure content; Vila-Vicosa et al., 2012) and, more often, minor structural modifications that may have an important impact on protein–protein association. Furthermore, the modulation of the ionizable side-chains’ charge controls the pattern of intermolecular electrostatic interactions established upon protein–protein association. Capturing the effects of pH on protein structure is particularly relevant in the case of β2m (e.g., wt and ΔN6) for which a slight (∼1 unit) decrease in pH is widely acknowledged to facilitate the onset of DRA (Giorgetti et al., 2005; Piazza et al., 2006; Esposito et al., 2012). The output of each CpHMD simulation is an ensemble of monomers representative of the intermediate state at a specified pH. Finally, in the third stage, these monomers are used in protein-protein docking simulations, based on a rigid-body protocol developed in house. Originally (Estacio et al., 2014; Loureiro et al., 2017), its cost function optimized the docking interface for shape complementarity by creating dimers with a minimal number of steric clashes and maximum number of contacts. The later version of the cost function considers steric interactions, hydropathic interactions, electrostatic interactions and hydrogen bonding (Loureiro et al., 2019), all considered key drivers of protein association (Meyer et al., 1996; Tsuchiya et al., 2006). The outcome of docking simulations is an ensemble of dimers (up to 2000 conformations) formed by monomers of intermediates under specific pH conditions. The dimers within each ensemble are eventually subjected to a classical MD protocol of structure relaxation to remove clashes and other structural errors. The relaxed ensemble of dimers is finally structurally analyzed to get information about the triggers of aggregation, i.e., the most likely regions initiating the process, and, at a finer level, the residues that will most likely establish a larger number of intermolecular interactions, acting therefore as aggregation hot-spots. It should be stressed that given the coarse-grained nature of the deployed cost function, the method should not be used to predict accurate structural models.

## Model Systems to Study β2m Aggregation

### Wild-Type Form

**FIGURE 3:**
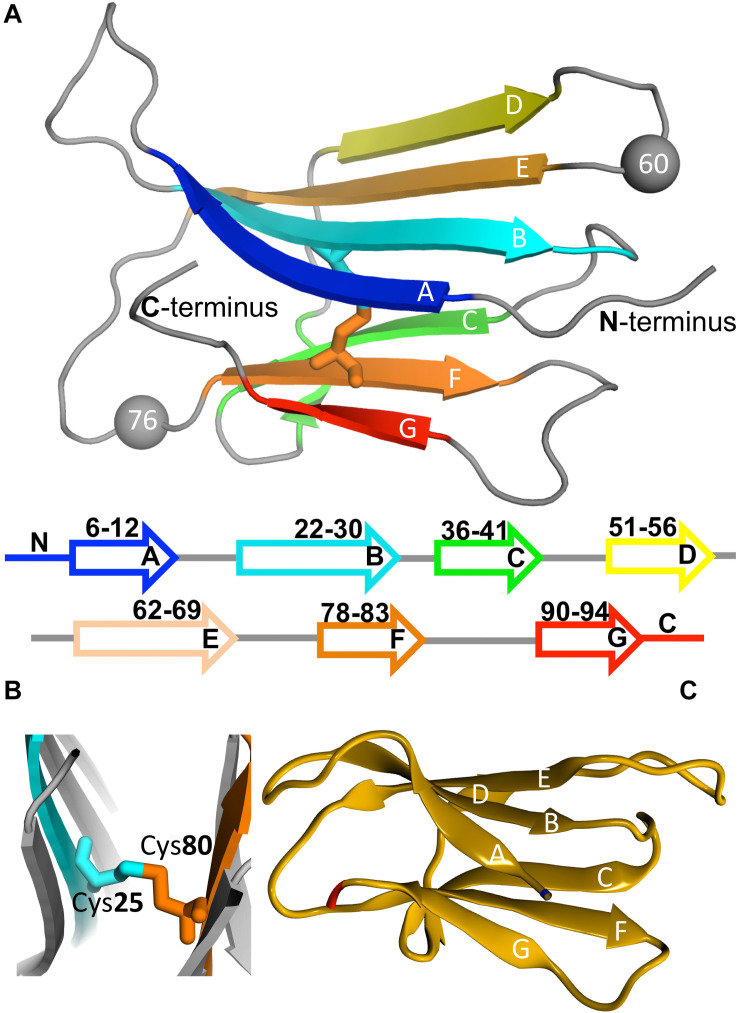
Protein beta-2-microglobulin (β2m). Three-dimensional structure of β2m highlighting the 7 beta-strands, organized in two beta-sheets (comprising strands A-B-E-D, and C-F-G, respectively), and the position of Trp60 (which is a key residue in aggregation) and Asp76, which is mutated to Asn in the D76N mutant (PDB ID: 4FXL) **(A)**. A cysteine bond links strands B and F **(B)**. Native structure of the ΔN6 structural variant lacking the N-terminal hexapeptide (PDB ID: 2XKU) **(C)**.

β2m is a 99 residue long protein of the immunoglobulin superfamily that constitutes the non-covalently bound light chain of class I major histocompatibility complex (MHC-1), assisting the efficient transport of nascent MHC-I chains to the surface of all nucleated cells (Becker and Reeke, 1985; Eichner and Radford, 2011; Esposito et al., 2012). The structure of the wild-type (wt) form comprises a classical β-sandwich fold with seven antiparallel β-strands (A through G) organized in two sheets of antiparallel β-strands, one comprising the strands A-B-E-D and the other comprising the strands C-F-G (Figure 3A). The native structure is stabilized by a disulfide bridge between the cysteine residues at positions 25 (at B strand) and 80 (at F strand) (Smith and Radford, 2001; Eichner and Radford, 2011; Esposito et al., 2012; Figure 3B), which has been regarded as fundamental in β2m fibrillogenesis. Indeed, when the Cys-Cys bond is not established there is a dramatic reduction in amyloid fibril formation (Hasegawa et al., 2003; Yamamoto et al., 2008). Another fundamental structural feature of β2m is the His31-Pro32 peptide bond in the BC loop, which adopts the thermodynamically unfavorable *cis*-isomer in the native structure and the *trans*-isomer when the protein partially or totally unfolds (Kameda et al., 2005; Esposito et al., 2012). High-resolution crystal structures of the wt form of β2m under physiological conditions, which are commonly used in molecular simulation studies, have been provided by Trinh et al. (2002) (PDB ID: 1LDS), and by Iwata et al. (2007) (PDB ID: 2YXF).

### Natural Variants

One intriguing feature of the wt form is its inability to aggregate *in vitro* in the absence of denaturants (e.g., trifluoroethanol), Cu^2+^, agitation, or amyloid seeds (i.e., pre-formed *ex vivo* amyloid fibrils), even under high protein concentration (100–200 μM) (Esposito et al., 2012). This limitation led researchers to consider natural and engineered variants that self-associate *in vitro* under physiological conditions as model systems of β2m aggregation. The structural variants ΔN6 (PDB ID: 2XKU) (Esposito et al., 2000; Ma and Nussinov, 2003; Fang et al., 2009b; Domanska et al., 2011; Eichner et al., 2011; Estacio et al., 2014; Hall et al., 2016), lacking the six N-terminal residues (Figure 3C), and ΔLys58 (Heegaard et al., 2002, 2005, 2006; Ma and Nussinov, 2003; Corlin et al., 2005), a cleaved form devoid of residue Lys58, are among the most frequently studied natural variants of β2m.

ΔN6 exhibits a high affinity for collagen (especially at pH 6.2; Giorgetti et al., 2005), being a major component (∼26%) of the *ex vivo* amyloid fibrils from DRA patients (Bellotti et al., 1998). Its incorporation into fibrils can be invoked to explain why this variant is not detected in the serum of DRA patients (Stoppini et al., 2005). As per today there is still no clear demonstration that the proteolytic cleavage of the N-terminal hexapeptide occurs pre-fibril assembly, and the possibility that it occurs afterward (Monti et al., 2002) compromises the use of ΔN6 as an appropriate model system to study β2m aggregation. Despite this limitation, the ΔN6 structural variant gained significant relevance as a model system to elucidate the mechanism of β2m aggregation, with some researchers actually proposing a leading role for this variant in β2m amyloidogenesis (Eichner et al., 2011).

ΔN6 has a high propensity to aggregate *in vitro* at physiological conditions, readily forming amyloid fibrils with agitation at neutral pH (Esposito et al., 2000; Bellotti et al., 2001; Eichner and Radford, 2009). It is thermally less stable than the wt form, and exhibits an increased structural flexibility that has been linked with its higher aggregation propensity (Eichner and Radford, 2009; Eichner et al., 2011). Indeed, studies *in vitro* based on NMR reported that loss of structure in strands A and C and part of strand B, and the consequent dislocation of strand D may be able to induce the formation of intermolecular contacts (Esposito et al., 2000; Bellotti et al., 2001) (i.e., to trigger aggregation). In line with these observations, an early computational study framed on MD simulations (Ma and Nussinov, 2003), concluded that ΔN6 is more structurally unstable than the wt form. In particular, the higher flexibility of strands B and E, leading to a larger separation between them, occurs concomitantly with the dislocation of strand D. Subsequent MD simulations predicted that the removal of the N-terminal hexapeptide, and the loss of the salt-bridge between residues Arg3 and Asp59 causes an increased solvent exposure of the K3 peptide (Ser20–Lys41), which ultimately leads to a greater mobility of strands B and E (Fang et al., 2009b), in line with previous results (Ma and Nussinov, 2003).

Contrary to ΔN6, the ΔLys58 structural variant is present in the blood of many DRA patients (Corlin et al., 2005), being, however, absent in the *ex vivo* amyloid fibrils (Giorgetti et al., 2007). Therefore, it is also not clear if ΔLys58 stands as an adequate model system to study β2m aggregation. Experiments *in vitro* showed that ΔLys58 extensively fibrillates upon seeding with β2m amyloid fibrils, and, in contrast to the wt form, forms high molecular weight non-fibrillar aggregates when incubated at physiological unseeded conditions (Heegaard et al., 2005). It is less structurally stable than the wt form (Heegaard et al., 2005, 2006), actually populating an intermediate state with increased affinity for Congo red under physiological conditions (Heegaard et al., 2002). ΔLys58 is also kinetically less stable than the wt form, exhibiting an unfolding rate that is one order of magnitude larger (Heegaard et al., 2005). Interestingly, MD simulations (Ma and Nussinov, 2003) reported that with the exception of an increased flexibility of the strands C’ and D, the conformational changes of ΔLys58 upon unfolding are similar to those of the wt form.

More recently, the Asp76Asn point mutant (PDB ID: 4FXL) was identified in a French family as the etiological agent of a hereditary systemic amyloidosis affecting visceral organs (Valleix et al., 2012). Indeed, all the heterozygous carriers of the mutation presented a rare form of systemic amyloidosis (autosomal dominant inheritance) characterized by the deposition of amyloid fibrils in several visceral organs (liver, kidney, spleen, and the heart) without the existence of any amyloid deposits in bones and ligaments (Valleix et al., 2012). This localization of the amyloid deposits is unexpected considering the known tropism of the wt form for the musculoskeletal system (Stoppini and Bellotti, 2015). Also surprising is the fact that Asp76Asn does not accumulate at high concentrations in the patient’s serum, and the absence of the wt form in the amyloid deposits (Valleix et al., 2012). The latter can be explained by the fact that the preferential accumulation of β2m on the surface of collagen only becomes significant for micromolar concentrations observed during hemodialysis, meaning that the sub-micromolar physiological concentrations observed in the Asp76Asn amyloidosis are not sufficient to induce the deposition of β2m fibrils in bones and ligaments (Stoppini and Bellotti, 2015).

In recent years, the Asp76Asn mutant became a valuable model system to study β2m aggregation (Valleix et al., 2012; Chong et al., 2015; de Rosa et al., 2015; Chandrasekaran and Rajasekaran, 2016; Leri et al., 2016; Loureiro et al., 2017; Le Marchand et al., 2018). The reason is twofold. First, upon agitation, the Asp76Asn mutant readily aggregates *in vitro* under physiological unseeded conditions (Valleix et al., 2012; Mangione et al., 2013). Secondly, since this mutant is the causative agent of a fatal hereditary systemic amyloidosis, it has a clear biological and biomedical relevance. The Asp76Asn mutant has, therefore, been the subject of several experimental and computational studies aimed at explaining its increased aggregation propensity and aggregation mechanism (Chong et al., 2015; de Rosa et al., 2015; Chandrasekaran and Rajasekaran, 2016; Leri et al., 2016; Loureiro et al., 2017; Le Marchand et al., 2018).

NMR measurements indicate that the solution structure of Asp76Asn does not differ significantly from that of the wt form (Valleix et al., 2012). MD simulations indicate that strand D is longer in the Asp76Asn mutant than in the wt form (due to an inward movement of residue Asp53), the DE-loop is more flexible, and there is local misfolding of all beta strands and turn regions due to the inability to form essential hydrogen bonds (Chandrasekaran and Rajasekaran, 2016). A study combining NMR and MD suggests that the Asp76Asn mutation reduces the mobility of the EF loop and of C, D, and E strands (Sakurai et al., 2019).

The negative charge loss occurring upon mutation is not associated with the higher aggregation potential of the Asp76Asn mutant, as originally hypothesized (de Rosa et al., 2015). However, the mutation causes a sharp decrease in thermodynamic stability, as quantified by a remarkable drop of 10°C in the melting temperature *T*_*m*_, which appears to be related with its aggregation potential. It has been speculated that the loss in thermal stability associated with the shear stress within the aqueous fluid of the extracellular matrix of visceral organs is sufficient to create a partially unfolded state with exposed hydrophobic patches. Furthermore, the interaction of the latter with the hydrophobic milieu of the extracellular matrix can cause a local accumulation of partially unfolded monomers that self-associate leading to a condition of supersaturation upon which soluble oligomers precipitate into insoluble aggregates. A detailed description of the mechanism triggering aggregation *in vivo* was proposed and discussed in detail in Stoppini and Bellotti (2015), but the exact role played by shear forces and hydrophobic interactions as triggers of aggregation *in vivo* remains to be established.

Interestingly, *in vitro* studies (de Rosa et al., 2017; Le Marchand et al., 2018) indicate that *any* mutation at position 76 causes a similarly large decrease in the protein’s thermal stability, and concomitant enhancement of the aggregation potential, without causing major structural alterations in the native structure. Position 76 within the EF loop has therefore been ascribed a critical role in determining the aggregation behavior of β2m.

### Engineered Mutants

Apart from the natural variants, some engineered mutants have been used to study β2m aggregation. These include the DE loop engineered mutants Asp59Pro, Trp60Cys, Trp60Val, and Trp60Gly (Kihara et al., 2006; Esposito et al., 2008; Ricagno et al., 2008, 2009; Santambrogio et al., 2010; Estacio et al., 2013; Gumral et al., 2013; Chong et al., 2015; Natalello et al., 2015; Camilloni et al., 2016; Narang et al., 2018), the AB loop engineered mutant His13Phe (Calabrese et al., 2008; Blaho and Miranker, 2009) and the BC loop engineered mutants His31Tyr (Rosano et al., 2004; Esposito et al., 2005) and Pro32Ala (Eakin et al., 2006; Blaho and Miranker, 2009).

There is clinical evidence that incidence of DRA is up to 50% lower among patients treated with Cu^2+^-free dialysis membranes. It has been suggested that the presence of this metal ion can play a role in amyloidogenesis of β2m under physiological conditions by destabilizing the native structure, thereby increasing the formation of partially folded intermediates that can prime the aggregation cascade (Eakin et al., 2006; Blaho and Miranker, 2009). Indeed, it is well known that ions bound to specific sites can profoundly alter protein stability and structure (Friedberg, 1974; Leal et al., 2012). The His13Phe, His31Tyr, and Pro32Ala mutants have been used to study the β2m aggregation mechanism in the presence of Cu^2+^.

The His13Phe mutant preserves the native state’s stability and the Cu^2+^ binding affinity of the wt form, but its early forming oligomers exhibit a higher stability than those of the wt (Calabrese et al., 2008). The His13Phe mutant oligomerizes preferentially into stable hexamers that have been structurally characterized (Calabrese et al., 2008), as we will discuss later. The Pro32Ala mutation converts the *cis* isomer of the His31-Pro32 bond (characteristic of the native state) to the *trans* isomer. Likewise, the Pro32Ala mutant was used to investigate the role of the isomerization of the His31-Pro32 bond in Cu^2+^-dependent formation of amyloidogenic conformations (Eakin et al., 2006). Additionally, double mutants containing the Asp59Pro substitution, plus the substitution of a histidine residue (e.g., His13Phe) were used to explore the role of individual imidazole side chains in Cu^2^ binding affinity, native state’s stability, and oligomerization mechanism (Blaho and Miranker, 2009). It was reported that Cu^2+^ binds primarily to residue His31 in the Pro32Ala mutant (Eakin et al., 2002; Verdone et al., 2002), which is the same binding site of the wt form, and that Cu^2+^ binding induces the formation of tetramers by the Pro32Ala mutant. As we will discuss later, in these tetramers interactions involving His51 play an essential role in stabilizing the oligomerization interfaces.

The His31Tyr mutant is structurally similar, but kinetically more stable than the wt form (Rosano et al., 2004; Esposito et al., 2005). However, the ensemble of conformers populated by this mutant is remarkably heterogeneous. In particular, there is a minor conformational state characterized by the detachment of the N terminal strand A from its native position, which is a structural modification frequently regarded as necessary to trigger β2m aggregation (Verdone et al., 2002; Estacio et al., 2014; Le Marchand et al., 2018). A similar conformational excursion was also observed for the ΔN6 variant in the context of DMD simulations of a full atomistic Gō model (Estacio et al., 2014).

Both the DE loop and strand D accommodate several aromatic bulky residues that become solvent exposed in the monomeric (free) form of β2m. In this region, residue Trp60 is of particular interest. This bulky amino acid, located at the apex of the DE-loop (Figure 2A), is highly conserved amongst vertebrates and plays a critical role in assisting the association of the β2m with the MHC-I heavy chain (Platt and Radford, 2009). These observations have motivated a series of *in vitro* experiments based on mutations of Trp60 and other nearby residues in order to establish their relevance for biological association of β2m.

Studies on three loop mutants (Trp60Gly, Trp60Cys, and Asp59Pro) (Esposito et al., 2008; Ricagno et al., 2008, 2009; Santambrogio et al., 2010) revealed that the Asp59Pro mutant aggregates more than the wt (Ricagno et al., 2008), being able to efficiently nucleate fibrillogenesis *in vitro* at physiological pH (7.4) (Ricagno et al., 2008). The Trp60Gly and Trp60Cys mutants, on the other hand, display a decreased amyloidogenic propensity relatively to the wt form at physiological pH, with Trp60Gly being the less aggregation prone (Esposito et al., 2008; Ricagno et al., 2008, 2009). The amyloidogenic behavior of all variants increases at acidic pH, with that of Trp60Cys becoming quantitatively comparable to the wt form (Ricagno et al., 2008). The native structure is broadly conserved across the three mutants, which only exhibit conformational changes in the DE loop. *In vitro* studies based on Trp fluorescence and circular dichroism (CD) indicate that the Trp60Gly mutant is conformationally more stable than the wt form, with the Trp60Val and the wt form showing similar conformational stabilities. The Asp59Pro mutant, on the other hand, shows a decreased conformational stability (Santambrogio et al., 2010). In line with this observation, recent MD simulations revealed a decrease in the average number of hydrogen bonds in the loop regions on Asp59Pro that enhances conformational flexibility (Narang et al., 2018). The melting temperature *T*_*m*_ of the model systems follows the same trend being 60.1°C (wt form) > 59.8°C (Trp60Cys) > 52.0°C Asp59Pro), and highlight a clearly more pronounced loss of thermal stability for the Asp59Pro mutant (Ricagno et al., 2008). The same qualitative results for *T*_*m*_ were replicated in DMD simulations of a full atomistic Gō model (Estacio et al., 2013). The different conformational and thermal stabilities have been invoked to rationalize the different aggregation propensities exhibited by the three mutants, an idea that is supported by the fact that the amyloid fibrils generated by the different DE loop mutants have the same general morphology and fibrillary architecture, suggesting conserved aggregation pathways across the three mutants (Natalello et al., 2015). The similar thermal and conformational stabilities and decreased aggregation propensities of both the Trp60Val and Trp60Cys mutants relative to the wt form indicate that the aromatic residues located in the DE loop, particularly at position 60, are essential in β2m aggregation (Calabrese et al., 2008; Platt et al., 2008). In line with this view, the Trp60Phe mutant conserves an aromatic residue at position 60 and aggregates to the same degree as the wt (Kihara et al., 2006).

Table 1 provides a summary of the model systems used to study β2m aggregation.

**TABLE 1:** Model systems used in the study of β2m aggregation together with its physical and structural properties.

Model system	Structural/Physical traits	More amyloidogenic than the wt?
ΔN6 (N-terminus)	Trans His31-Pro32 bond/Low structural stability	Y
D76N (EF loop)	Longer D strand/Higher flexibility of DE loop; Low *T*_*m*_	Y
Δ58K (DE loop)	Increased flexibility of strands C’ and D/Low kinetic stability	Y
H13F (AB loop)	Conserved native state’s stability and Cu^2+^ binding affinity	N
H31Y (BC loop)	Higher kinetic stability	Not available
P32A (BC loop)	Trans His31-Pro32 bond; hydrophobic core repacking	N
D59P (DE loop)	Less hydrogen bonds in loop regions/Low *T*_*m*_	Y
W60G (DE loop)	Geometric relaxation of DE loop/Higher structural stability	N
W60C (DE loop)	Geometric relaxation of DE loop/Similar (but slightly lower) *T*_*m*_	N

## Early Phase of β2m Aggregation

### Aggregation-Prone Monomers of the wt Form

It has been proposed that the formation of one or more folding intermediate states, resulting from structural fluctuations of the native conformation, is necessary to initiate β2m aggregation (Armen and Daggett, 2005; Rennella et al., 2010; Eichner and Radford, 2011; Franco et al., 2017). These intermediates may expose aggregation-prone patches that are normally buried in the native structure as a result of local (Chiti and Dobson, 2009) and/or global unfolding events, a common trigger of the aggregation mechanism of several globular proteins (Liu et al., 2000; Jahn et al., 2006; Eichner and Radford, 2011).

Seminal studies by Chiti and co-workers identified two intermediate states in the folding pathway of the wt form under nearly physiological conditions (pH 7.4, 30°C): an ensemble of partially folded conformers (termed I_1_) with substantial elements of non-random structure that forms from the denatured state, and a low-populated intermediate state I_2_ forming (directly or indirectly) from I_1_ in the slower phase of folding, representing from ∼5% (Jahn et al., 2006) up to ∼15% (Chiti et al., 2001a) of the equilibrium population and presenting a more consolidated protein core. Compared to the native state, this conformer has a lower amount of beta sheet content and a more exposed and unstructured hydrophobic core (Chiti et al., 2001a). It presents a fivefold increase in its propensity to aggregate in the presence of preformed amyloid fibrils when compared with the native state (Chiti et al., 2001a). This species is generally designated by I_T_ due to a non-native *trans*-isomerization of the His31-Pro32 peptide bond (Kameda et al., 2005). The latter, which is considered a structural hallmark of this intermediate state, results from a complex conformational transition that involves the repacking of the hydrophobic core (particularly, Phe30, Phe56, Trp60, Phe62, Tyr63, Tyr66, Phe70, and Trp95) (Corazza et al., 2004; Kameda et al., 2005; Eakin et al., 2006; Calabrese et al., 2008), as well as a conformational rearrangement involving the N-terminus, BC and DE loops and their adjacent strand extremities, as observed with NMR (Corazza et al., 2004, 2010; Rennella et al., 2010). In line with these experimental observations, molecular simulations framed on MD predicted that, despite maintaining a native-like tertiary structure, the I_T_ intermediate has an increased conformational flexibility (specially of the AB, BC, and DE loops) (Torbeev et al., 2015), a disordered D strand, the hydrophobic core residues surrounding these structural elements exposed to the solvent (Chong et al., 2015), and a disruption of a network of hydrogen bonds involving residues His31, Pro32, and residues located in the N-terminus and in the FG loop, leading to a rearrangement of these regions (Fogolari et al., 2011; Table 2).

**TABLE 2:** Structural features of the intermediate I_T_ and methodologies used to determine it.

Structural hallmark of I_T_	Methodology
Non-native *trans* isomerization of His31-Pro32	NMR
Repacking of the hydrophobic core	NMR and MD
Rearrangement of N-terminus, BC and DE loops (+ adjacent strand extremities), & FG-loop	NMR and MD
Lower amount of β-sheet content	CD
Higher aggregation propensity	Thioflavin T fluorescence and dynamic light scattering

Some studies proposed a role for Cu^2+^ ions in the process of conformational conversion leading to I_T_, namely in the isomerization of the His31-Pro32 bond (Eakin et al., 2006; Calabrese et al., 2008) upon Cu^2+^ binding to His31 imidazole ring, and in a conformational change of Phe30 side-chain (Calabrese et al., 2008). These structural changes eventually lead to a reorganization of the aromatic side-chains of the BC and DE loops, giving rise to an alternative well-defined hydrophobic core (Eakin et al., 2006; Calabrese et al., 2008).

Daggett and colleagues used unfolding MD simulations (at 498K) to explore the conformational space of the wt form (Armen et al., 2004; Armen and Daggett, 2005). They reported the existence of intermediate states characterized for having α-sheet secondary structure in regions of the polypeptide chain that map to amyloidogenic peptides, suggesting that this structural motif may play a role in driving the formation of prefibrillar amyloidogenic oligomers.

### Aggregation-Prone Monomers of ΔN6

Studies based on NMR measurements indicate that at pH 7.5 and 25°C ΔN6 largely populates (90%) a conformational species that is structurally similar to the folding intermediate I_T_: it retains the native fold and preserves the *trans* isomerization of Pro32 while simultaneously undergoing a major reorganization of several side chains (particularly of Phe30 and Phe62) within the hydrophobic core (Eichner and Radford, 2009; Eichner and Radford, 2011; Eichner et al., 2011). Eichner et al. (2011) proposed that the ΔN6 structural variant is responsible for inducing the transition of the native full-length molecule to a fibril-competent conformation through a prionlike templating mechanism. According to this hypothesis, molecular collisions between ΔN6 and the wt form cause transient binding between the two species (most likely through residues of the ΔN6 form located in the BC, DE, and FG loops, and residues from the BC and DE loops of the wt form) (Karamanos et al., 2014). This interaction increases the conformational dynamics of the N-terminal strand of the full length protein, which, in turn, leads to a highly dynamic configuration of Pro14 in the AB loop (Eichner and Radford, 2011; Eichner et al., 2011); the latter has been shown to induce an alternative conformation of the full length protein in which the set of hydrogen bond interactions between strands A and B is critically impaired (Rosano et al., 2004). These events are critical because the hydrogen bonding between strands A and B together with the native conformation adopted by the N-terminal strand are essential for keeping a low equilibrium concentration of I_T_. Indeed, binding of ΔN6 to wt form disrupts important interactions between the N-terminal strand and the BC loop, facilitating the isomerization of the His31-Pro32 peptide bond and the formation of the amyloidogenic intermediate state (Eichner and Radford, 2011; Eichner et al., 2011). This prionlike hypothesis was severely criticized and challenged by Mangione et al. (2013), who reported that the wt form does not fibrillate with monomeric ΔN6 but rather with preassembled fibrils of ΔN6.

The ΔN6 variant has increased conformational dynamics being able to populate rare conformers capable of nucleating and elongating amyloid fibrils (Eichner et al., 2011). Interestingly, a slight decrease in pH (from 7.2 to 6.2), which occurs in joints during inflammation (Giorgetti et al., 2005; Piazza et al., 2006), may increase the population of these rare conformers by destabilizing the ΔN6 conformation through the protonation of His84 (close to Pro32) (Eichner et al., 2011). Indeed, this histidine undergoes a large pKa shift from 4.1 to 5.9 from the full-length to the truncated species, substantially increasing its protonation at pH 6.2 (Esposito et al., 2012).

**FIGURE 4:**
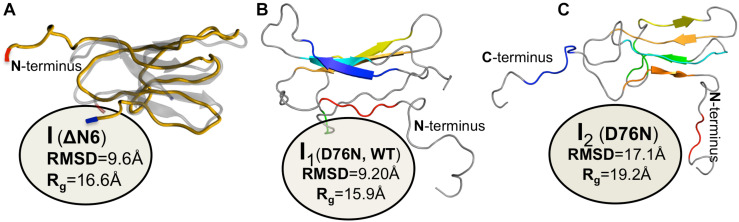
Simulated monomers of β2m. Three dimensional structure of three aggregation prone monomeric states predicted by a full atomistic Gō model, used to explore the folding space of ΔN6 **(A)** and D76N mutant **(B,C)**. The intermediate populated by ΔN6 features an unstructured N terminal region **(A)**, while those populated by D76N display the C-terminus **(B)**, or both termini unstructured **(C)**.

A simulated intermediate state for folding and aggregation of ΔN6 that preserves the nativelike core (extending from residue 21 to residue 94), conserves the *trans* isomerization of Pro32 characteristic of I_T_, and features an unstructured strand A that detaches significantly from the core was recently reported by Estacio et al. (2014) in the context of DMD simulations of a full atomistic Gō model (Figure 4A). It is possible that such an intermediate state, termed ΔN6-I, is one of ΔN6’s rare conformers, representing a conformational excursion of the I_T_ intermediate. Docking simulations predict a central role of the N-terminal region (comprising strand A and the AB loop) in dimerization. Interestingly, CpHMD simulations predict that strand A becomes maximally detached from the core region at pH 6.2, which results in higher aggregation propensity at this slightly acidic pH. Since this is the pH of inflamed joints, this prediction provides a mechanistic insight into the higher aggregation potential observed *in vitro* at pH 6.2, while simultaneously contributing to rationalize the pathogenesis of DRA. The association of an unstructured/detached strand A with the onset of fibrillogenesis in β2m was originally proposed by Verdone et al. (2002), and subsequent studies have linked this structural trait with acidic pH (McParland et al., 2000, 2002; Corazza et al., 2004; Mukaiyama et al., 2013b) or Cu^2+^ binding (Morgan et al., 2001; Verdone et al., 2002; Calabrese et al., 2008).

### Aggregation-Prone Monomers of the Asp76Asn Mutant

The Asp76Asn mutant abundantly populates (∼25%) an intermediate state that is structurally compatible with I_T_ under physiological conditions (Mangione et al., 2013). This concentration represents a fivefold increase in the equilibrium population of this species relative to that populated by the wt form, and correlates with the amyloidogenic character of β2m. MD simulations indicate that the Asp76Asn I_T_ intermediate has an enhanced β-sheet forming propensity in its disordered strand D and a ∼2% increase in SASA of the hydrophobic residues (Chong et al., 2015). Its solvation free energy is higher in comparison with the I_T_ intermediate populated by the wt form (Chong et al., 2015), in line with the higher aggregation potential of the Asp76Asn mutant.

The intermediate state I_T_ populated by Asp76Asn represents a highly dynamic structure (Stoppini and Bellotti, 2015), and conformational excursions to conformers with an even higher aggregation potential should not be ruled out. Recently, Loureiro et al. (2017) explored the folding space of Asp76Asn with DMD simulations of a full atomistic Gō model. The model predicts that the mutant populates two intermediate states with aggregation potential. Structurally, they share a well-preserved core but differ significantly in the terminal regions. The first intermediate, I_1_, which is also populated by the wt form, features the C-terminus unstructured (Figure 4B). The second intermediate, exclusively populated by Asp76Asn, features two unstructured termini, and is the most aggregation-prone species (Figure 4C). A posterior study that combined *in crystallo* with *in silico* data (Le Marchand et al., 2018) reported that Asp76Asn sparsely populates a highly dynamic conformation that exposes very aggregation prone regions as a result of a loss of beta structure at the N and C terminal strands resulting from the disruption of a large network of electrostatic interactions involving the termini and the EF loop. Conformational dynamics in this species is especially enhanced relatively to the wt form in the EF loop (where the mutation is located) and in residues located at the end of strand A. The authors associated the higher aggregation propensity of Asp76Asn to the destabilization of its outer strands, in line with predictions from DMD simulations (Loureiro et al., 2017). This conformational state is topologically compatible with the most aggregation prone intermediate predicted by the native-centric model of Loureiro et al. (2017), and highlights the relevance of unstructured termini as potential triggers of aggregation. Indeed, it is possible to speculate that the shear forces present in the extracellular fluid under physiological conditions may be enough to further unfold the intermediate’s termini and induce amyloid formation *in vivo*.

### Aggregation-Prone Monomers of DE Loop Mutants

Estacio et al. (2013) studied the folding transition of the wt form, Asp59Pro and Trp60Cys mutants with a full atomistic Gō model and DMD simulations. A common intermediate state was found for the three variants featuring a well-preserved core region (strands B-F), and two unstructured termini. About 40% of the intermediate’s hydrophobic residues have a SASA that is (40 times) larger than in the native state, suggesting an aggregation potential for this species. Interestingly, the size of the intermediate’s population increases with decreasing thermal stability, being therefore larger for the less thermally stable and most aggregation prone Asp59Pro mutant. The free energy surfaces representing the folding space of wt form shown that this intermediate forms from the denatured state through the crossing of a small free energy barrier, indicating that it can represent the I_1_ intermediate reported by Chiti et al. (2001a, b), or some conformational excursion of I_1_ on the way to the I_T_ intermediate. Interestingly, the structural characteristics of the identified intermediate resemble that of a molten globule state of β2m identified at pH 4.0 (Mukaiyama et al., 2013a, b), whose structure consists also of a stable and compact core comprising strands B, C, D, E, and F and intervening loops, and in highly unstructured termini. Additionally, PROPKA predictions of pKa suggest that the population of the intermediate state may be favored at pH 4.0 as the charge of the native protein becomes more positive at this pH, which indicates that the identified intermediate could be the molten globule state identified *in vitro*.

### Dimers

The dynamic and transient nature of the oligomeric states formed in the beginning of the aggregation pathway poses a challenge to the experimental investigation and *in vitro* structural characterization with atomic detail of these species. Despite these difficulties, studies conducted by several research groups indicate that the first phase of the β2m aggregation mechanism is the dimerization of aggregation prone monomers (Fabian et al., 2008; Eichner and Radford, 2009; Rennella et al., 2013; Halabelian et al., 2015).

One of the first studies addressing the formation of dimers by β2m was a theoretical investigation that combined sequence and structural conservation analysis with docking simulations (Benyamini et al., 2003, 2005). It predicted a model for β2m fibrillogenesis in which monomers with native-like conformations associate via head-to-tail pairwise interactions originating a new inter-monomer beta sheet formed by strand B of one monomer and strand D of the other. The establishment of a cluster of aromatic interactions involving Phe56 from one monomer, and Phe30, His31, and Trp60 from the other monomer contributes to dimer stability. The importance of Phe56 and Trp60 in self-association of β2m was also reported in a later study based on MD in explicit solvent, which explored the pattern of intermolecular interactions establishing within an ensemble of 27 monomers representative of the native state of β2m during 5-ns at physiological temperature (Fogolari et al., 2007). Within the simulation timescale the most frequently found pattern of interaction between the native monomers involved a head-to-head contact arrangement in which DE loop residues (Phe56, Lys58, Asp59, Trp60), N-terminal (Ile1, Arg3), and C-terminal (Arg97, Asp98, Met99) residues from both monomers establish the most ubiquitous intermolecular contacts, while hydrophobic interactions involving Trp60, Phe56, and Ile1 lead to the most stable (in the timescale of the simulation) dimer conformations. A head-to-head dimer arrangement for the wt form involving the same apical regions was also proposed in a NMR study (Rennella et al., 2013), and heterodimers of the wt form and ΔN6 appear to assemble through head-to-head interactions involving the BC, DE, and FG loops (ΔN6), and the BC and DE loops (wt form) (Karamanos et al., 2014).

**FIGURE 5:**
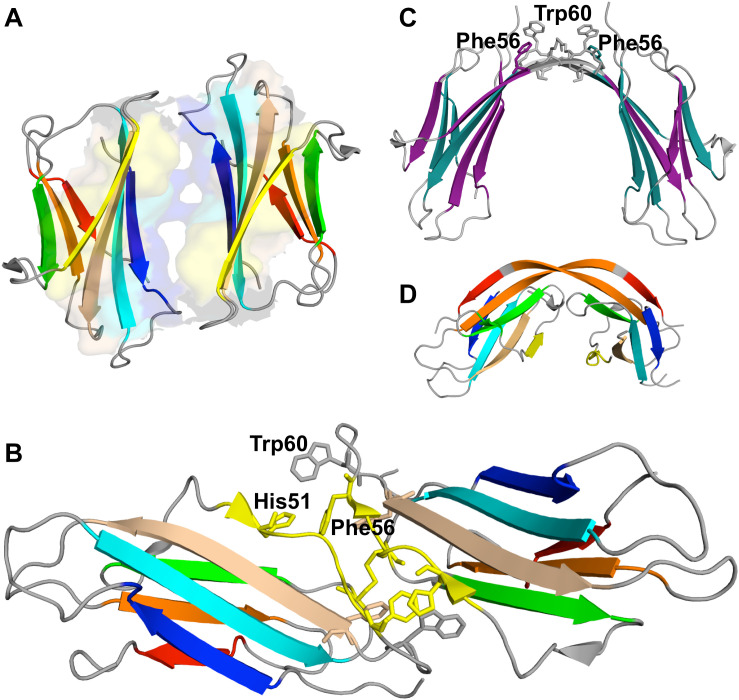
Dimers of β2m. P32A dimer featuring an anti-parallel eight-stranded beta-sheet ABED-DEBA (PDB ID: 2F8O), which is mediated by antiparallel interactions of two complete D strands **(A)**, DIMC33 formed by two S33C monomers linked by a disulfide bond at position 33 (PDB ID: 4R9H), which exhibits the DD strand interface **(B)**, domain-swapped dimer of the wt formed under reducing conditions, where strands E-F-G are exchanged between monomers (PDB ID: 3LOW); the residues forming the hinge loop, which contains amyloidogenic peptide segments, is highlighted **(C)**. Domain-swapped dimer of the ΔN6 variant, which exchanges strand G (PDB ID: 2 × 89) **(D)**.

A seminal experimental study investigating β2m dimerization was based on the single point mutant Pro32Ala (Eakin et al., 2006), which adopts a preferential conformer that may be considered a structural mimic of the intermediate I_T_ in the presence of Cu^2+^ ions. The crystal structure of the Pro32Ala dimer (PDB ID: 2F8O) indicates that self-association is driven by antiparallel interactions between the D strands of the two interacting monomers, yielding an eight-stranded ABED-DEBA beta sheet and forming a buried hydrophobic patch composed of Phe30, Leu54, Phe56, Phe62, and Tyr63 (Figure 5A). This elongated dimer conformation can further oligomerize either by head-to-head (through the D-strands) or tail-to-tail (through the A strands) interactions, and is compatible with the typical dimensions of amyloid fibers. However, since the Pro32Ala mutant does not form fibrils *in vitro* (Mendoza et al., 2010) one should not rule out the possibility that the crystalized dimers contain structural features that prevent further oligomerization, and, if this is the case, it does not provide an accurate structural model of the dimer formed by the wt monomers.

A posterior study, which combined experimental and computational methods (including covalent labeling, MS and docking simulations) to explore the self-association of wt monomers in the presence of copper ions, strengthened the importance of antiparallel stacking of the ABED beta sheets as a structural motif in β2m dimer formation, but excluded the possibility of a dimer interface stabilized by intermolecular interactions between the D strands. Instead, it reported that dimer stabilization is driven by hydrophobic and electrostatic interactions between residues in the AB loop and residues in the DE loop (Arg12 with Tyr63 and Lys19 with Asp59) (Mendoza et al., 2010). A previous study by the same group (Srikanth et al., 2009) proposed that the establishment of the stabilizing salt-bridge between Asp59 and Lys19 occurs as a result of repositioning of Asp59 following Cu^2+^ binding. Analogously, Cu^2+^ binding also dislocates Arg3 at the N terminus, allowing it to establish a stabilizing salt bridge with Glu16 in the AB loop.

The role of an antiparallel dimerization interface mediated by the association of the BC loop, strand D, DE loop and strand E—termed the DD strand interface—was investigated by analysing the oligomerization of disulfide-linked homodimers of β2m cysteine mutants Ser20Cys (AB-loop), Glu50Cys (D-strand) (Colombo et al., 2012), and Ser33Cys (BC-loop) (Halabelian et al., 2015). These engineered dimers are respectively termed DIMC20, DIMC50, and DIMC33 (Figure 5B). The structural analysis of dimer’s crystals indicates that the main residues involved in the stabilization of the DD interface are His31, Asp34, Phe56, and Trp60, in line with results reported in other studies (Benyamini et al., 2003, 2005; Eakin et al., 2006; Fogolari et al., 2007; Mendoza et al., 2010). Interestingly, another dimer interface stabilized by aromatic residues Tyr10, Tyr26, and Tyr63 has been reported exclusively for the DIMC33 dimer (Halabelian et al., 2015). The DCIM20 and DCIM50 dimers are able to induce the aggregation of the wt form in unseeded conditions, suggesting an important role for the DD interface in β2m amyloidogenesis.

Recently, we investigated the interfacial region of dimers of the full-length wt form by performing extensive molecular docking simulations (Estacio et al., 2014) of monomers equilibrated in CpHMD simulations at physiological and slightly acidic pH 6.2. We found that, independent of pH, dimerization of the native monomer is majorly driven by the DE loop (especially residues 56–60), and that in the best packed dimers the DE loop directed interactions involve preferentially the aromatic rich regions of the BC and DE loop of the other monomer. The latter, which are both located on the same side of the monomer, become unavailable to interact with other monomers, and subsequent oligomerization appears to be restricted to the potentially adhesive residues located on the EF loop (e.g., Phe70 and Tyr78) and in the C terminus (Trp95). These observations indicate that a dimerization mechanism driven by head-to-head loop coupling leads to stable soluble dimers, which may represent a dead-end for aggregation, in line with the little amyloidogenic character of the wt form in physiological conditions.

At pH 7.2 the homodimers of the intermediate state populated by ΔN6 (Figure 4A) associate through the DE loop and BC loop, and, to a lesser extent, via the FG loop and the C terminus. When the pH is lowered to 6.2 the DE loop conserves its importance, and the interactions involving the FG loop gain relevance. More interesting, however, was the finding that the N terminal region (comprising the A strand and AB loop) became an important adhesion zone. In particular, our simulations predict that ΔN6-I uses its unstructured strand A to recruit another monomer by interacting with its DE, EF or FG loop. This interaction pattern leaves the other monomer’s strand A available to oligomerize further by recruiting another monomer. Such dimerization interface is stabilized preferentially by interactions involving Trp60, Phe30 and Tyr10, along with His13, His31 and His84. The histidines are particularly interesting aggregation hot spots. They interact mostly with Trp60, which displays an aromatic side chain. The protonation of histidines (whose pKa is ∼6.0) at the slightly more acidic pH 6.2 of the inflamed joints implies that a cation-π-interaction will establish involving the positively charged imidazole ring of the histidine residue and the negatively charged indole π-electron could in the aromatic amino acid. The increase in dimer stability resulting from these interactions (up to 2 kcal/mol per interaction) can contribute to dimer stabilization at slightly acidic pH (Estacio et al., 2014).

The importance of the N terminal region in β2m aggregation was highlighted in a study that investigated the effect of the Cys25-Cys80 disulfide bond on β2m oligomerization by means of discrete MD simulations of a symmetrized structure-based Gō potential (Chen and Dokholyan, 2005). It was found that under oxidizing conditions (i.e., when the intramolecular disulfide bond is formed), the wt form of β2m originates domain-swapped dimers in which the two monomers keep most of the native fold but exchange their N-terminal segments (Chen and Dokholyan, 2005). A structurally different dimer of β2m, also formed on the basis of a domain swapping mechanism, was found in experiments *in vitro* (PDB ID: 3LOW) (Liu et al., 2011). In this dimer, which formed slowly under physiological conditions, strands E, F and G are exchanged between the two monomers (Figure 5C).

We investigated the dimerization interface of the Asp76Asn mutant through docking simulations at physiological and acidic pH 5.2 (Loureiro et al., 2019). We focused on the two intermediate states featuring one (I_1_) and two (I_2_) unstructured termini (Figures 4B,C), and considered interfaces formed by homo- and heterodimers. We found that the DE loop and EF loop behave as adhesion zones at pH 7.2 being, however, more important in the association of the I_2_ intermediate. It is possible that the detachment of both the N and C terminal regions from the core in the I_2_ intermediate state facilitates (and fosters) the movement of the DE and EF loops, in line with observations reported in Le Marchand et al. (2018). The leading hot spot residue at physiological pH is clearly Trp60 (DE loop), but its role as an interaction hub sharply decreases at acidic pH. In this case, the dimerization of I_2_ is majorly triggered by interactions involving Arg3 (N terminus), followed by two clusters of residues located on the DE-loop and adjoining D strand (His51, Phe56, and Trp60) and, to a lesser extent, on the EF loop and adjoining E strand (Tyr67, Phe70, and Lys75). The participation of Arg3 (N terminus), Tyr10, and Arg12 (A strand) in the association pattern of homo- and heterodimers, particularly, at physiologic pH, is also noteworthy. Under acidic pH 5.2, the C terminus gains relevance as an adhesion zone in the heterodimers and more strikingly in the I_1_ homodimers, possibly as a result of the detachment of the C terminus upon protonation of Hys84 (FG loop). The AB loop is also an important structural element in dimerization of I_1_, establishing preferential interactions with the EF loop and AB loop of the other monomer, as well as with the C-terminus. At pH 5.2, I_1_ monomers associate mainly through Trp95 and Arg97 (C-terminus), followed by His13 and Lys19 (both pertaining to the AB loop). The former are also leading hot spots in dimerization of the heterodimers, where His51 (D strand) also acts as hot spot, because of its increased protonation at acidic pH (pKa∼6.5). To the best of our knowledge the possibility that Lys75 and Trp95 are able to nucleate aggregation in β2m has never been acknowledged either in simulations or experiments *in vitro*.

Overall our detailed computational studies (Estacio et al., 2014; Loureiro et al., 2017, 2019) indicate an essential role in dimerization for the C and N terminal regions (Asp76Asn and ΔN6), as well for the BC (ΔN6 variant), DE (Asp76Asn and ΔN6), and EF (Asp76Asn mutant) loops in dimerization. The terminal regions are more relevant under acidic conditions while the BC, DE and EF loops gain importance as structural interface elements at physiological pH. The participation of the N terminus (Thr4 and Pro5) and BC loop (His31) was recently reported in interfaces of dimers of Asp76Asn obtained by docking simulations (Cantarutti et al., 2017; Brancolini et al., 2018).

### Trimers and Pentamers

The formation of trimers (Smith et al., 2006, 2010, 2011) and pentamers (Smith et al., 2010, 2011) was reported in studies based on MS that analyzed the early stage of β2m fibrillogenesis under acidic conditions. However, no structural model has been proposed for the pentamer so far, and there is only one experimental article in the literature proposing a structural model for a β2m trimer, which is based on an integrative approach comprising cross-linking, MS, Monte Carlo and MD simulations (Hall et al., 2016). The trimer is comprised of two structural units: the ΔN6 domain-swapped dimer reported in Domanska et al. (2011) (PDB ID: 2X89) (Figure 5D), and a native ΔN6 monomer. A structural characterization with atomic detail was, however, not provided. A structural model for a trimer for the wt form obtained under reducing conditions (i.e., in which the disulfide bond is not formed) was proposed in Chen and Dokholyan (2005). In the trimer, the monomers are significantly unfolded in extended conformations, which stack in parallel to each other, forming inter-chain beta-sheet structure. In the context of this model it was proposed that the formation of amyloid fibrils could result from the stacking of trimers along the fibril axis.

### Tetramers and Hexamers

*In vitro* experiments based on dynamic light scattering indicate that in the presence of Cu^2+^, oligomerization of the wt form proceeds exclusively through the formation of even-numbered oligomers (i.e., soluble tetramers and hexamers) resulting from the association of dimeric units (Antwi et al., 2008), which emphasizes the importance of dimers in Cu^2+^ induced β2m aggregation. In contrast, β2m amyloid formation under acidic pH proceeds via odd- and even-numbered oligomers (ranging from dimers to tetradecamers), which suggests that oligomerization proceeds via addition of monomers (Smith et al., 2011; Marcinko et al., 2019). Interestingly, early studies on the structural characterization (via cryo-EM) of amyloid fibrils obtained from monomers of the wt form under acidic pH 2.5 indicate that the basic assembly units of the fibril protofilaments are tetramers obtained by a dimer-of-dimers arrangement (White et al., 2009). In contrast, a recent study suggested that amyloid formation for the wt form under acidic pH proceeds by the parallel in-register stacking of monomers mediated by hydrogen bonds between backbone atoms in the beta strands and by π-stacking interactions between the aromatic residues Phe22, Tyr26, Phe30, Phe56, Trp60, Phe62, Phe70, and Tyr78 (Iadanza et al., 2018).

A structural model for the tetramer obtained under physiological conditions in the presence of Cu^2+^ (Mendoza et al., 2011) reported an interface formed by strands D of one dimer and strands G of the other dimer, stabilized by four salt bridges (Glu50-Arg97, His51-Asp96, Asp53-Lys91, and Asp53-Ly94). A recent study by the same group (Marcinko et al., 2019), combining experiments *in vitro* with molecular simulations, emphasized the importance of conformational heterogeneity induced by copper binding, with Cu^2+^-dependent β2m aggregation proceeding via the formation of different tetrameric species, some being Cu^2+^-bonded and others Cu^2+^-free. Nevertheless, some key interactions appear to be conserved in the central interface of the different tetramers, namely, a cation-π interaction between His51 and Phe56, the hydrophobic interaction between Leu54 and Leu54, and a salt bridge between Glu50 and Lys58. The central interface of the Cu^2+^-free tetramers is specifically stabilized by a salt bridge between His31 and Asp34, and a cation-π interaction between His51 and Trp60.

**FIGURE 6:**
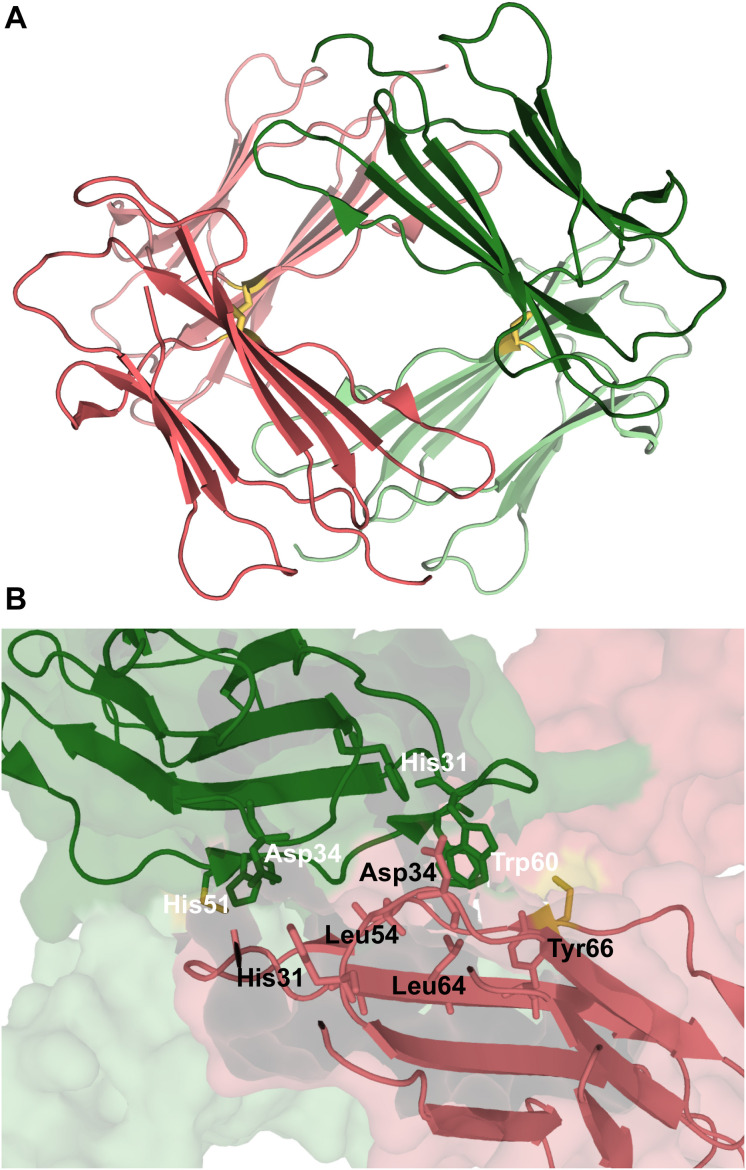
Tetramer of β2m. Three-dimensional structure of the tetramer **(A)**, formed by two disulfide linked homodimers at Cys50 (PDB ID: 3TM6). In this representation the homodimers are colored green and pink, respectively. The DD strand interfacial region in **(B)** is the largest found in this tetramer being stabilized by hydrogen bonds between His31 and Asp34, and hydrophobic interactions involving Trp60, Leu54, Leu64, and Tyr66.

A different model for the tetramer of β2m was proposed based on the structural characterization of tetramers formed by the disulfide-linked covalent homodimers DIMC20 (PDB ID: 3TLR) and DIMC50 (PDB ID: 3TM6) (Colombo et al., 2012). The covalent bond between the mutated cysteine residues of the two monomers constraints the morphology of the dimer, which may hamper or facilitate further oligomerization, while allowing to evaluate the importance of the DD interface (BC loop, D strand, DE loop, and the E strand) in amyloidogenesis. It was found that the dimers assemble into tetramers that largely conserve the DD interface, and eventually fibrillate into amyloids. In the case of the DIMC50 tetramer (Figure 6A) the DD interface is stabilized by hydrophobic interactions involving Phe56 and Trp60 from one subunit and Leu54, Leu64 and Tyr66 from the other, together with hydrogen bonds between His31 of one subunit and the Asp34 of the other (Figure 6B). In the DIMC20 tetramer Phe56 is solvent exposed, but overall the tetramer’s interface is similar to that of the DIMC50 tetramer. The D strand, and possibly also the neighboring DE loop, were also found to participate in the interfacial region of a Cu^2+^-bounded tetramer of the Pro32Ala mutant (Blaho and Miranker, 2009).

Synthetic peptides incorporating the amyloidogenic heptapeptide sequence β2m_63__–__69_ pertaining to strand E assemble into beta-sheets that subsequently associate into hexamers (which are trimers of dimers), octamers (which are tetramers of dimers) and dodecamers (with two trimer subunits surrounded by three pairs of beta-sheets) (Spencer et al., 2015).

The fact that ΔN6 and Asp76Asn mutants aggregate rapidly in physiological pH conditions may be taken as an indication that the amyloid fibrils formed by these variants are different from those formed by the wt form. In line with this hypothesis, the interfacial region of a tetramer of the ΔN6 variant formed by the domain-swapped dimers (Domanska et al., 2011) was found to comprise the FG loops of each dimer (i.e., the hinge regions of the originating domain-swapped dimers), being stabilized by hydrogen bonds involving residues Thr86, Ser88, and Gln89 (Hall et al., 2016).

Recently, we carried out the very first computational study that investigated the structure of tetramers formed by the Asp76Asn mutant (Loureiro et al., 2019). We focused our analysis on dimers formed by the I_2_ intermediate state, which features the most aggregation prone traits. Our study, based on extensive docking simulations, indicate that the N-terminus together with the DE loop are the most important adhesion zones in the tetramer, whose interfacial region is stabilized by intermolecular interactions involving Trp60 (DE loop), Arg3 (N terminus), Phe56 (D strand), Tyr10 (A strand), and, to a lesser extent, Lys58 (DE loop) and Arg97 (C terminus).

**FIGURE 7:**
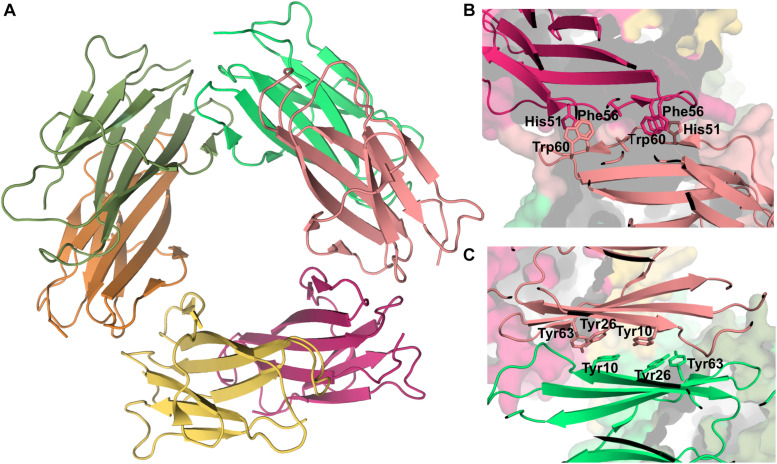
Hexamer of β2m. Three dimensional structure of the hexamer formed by the H13F mutant in the presence of Cu^2+^ (PDB ID: 3CIQ) **(A)**. The hexamer results from the association of dimers that form an interface mediated by D-strands from adjacent chains stabilized by hydrogen bonds between Leu54, Asp34, and His31 (not shown) and hydrophobic interactions involving Phe56 and Trp60 from one chain and His51 and Asp34 from the other **(B)**. The other interface is mediated by the stacking of the ABED sheet from one chain onto the ABED sheet of an adjacent monomer in an antiparallel arrangement and stabilized by successive interactions involving Tyr residues **(C)**.

Experimental data based on X-ray crystallography indicates that the hexamer formed by the His13Phe point mutant of β2m in the presence of Cu^2+^ (PDB ID: 3CIQ) is structurally characterized for being an association of three dimers, having two distinct interfaces (Figure 7A). The dimer interface (with a surface area of 1340 Å^2^) forms as a result of the displacement of residues Phe56 (3.1 Å) and Trp60 (8.0 Å) upon Cu^2+^ binding, being stabilized by intermolecular interactions between the D strands of each interacting monomer (Figure 7B). Particularly, by hydrogen bonds between the Leu54 main chain of one monomer and the Asp34 and His31 side chains of the other monomer, and by hydrophobic interactions between Phe56 and Trp60 of one monomer and the non-polar atoms of His51 and Asp34 of the other monomer. The inter-dimer interfacial region is considerably larger than the dimer interface (1950 Å^2^), and results from the antiparallel stacking of the ABED sheets from two adjacent dimers (Calabrese et al., 2008), with each strand approximately opposing its counterpart from the other dimer (e.g., A:A, B:B) (Figure 7C). The core of this interface comprises both aromatic and polar interactions between tyrosines 10, 26 and 63, and includes also residues Ile1, Arg3, and Phe30 of the Cu^2+^ binding site from each dimer. These residues are displaced from their positions in the wt Cu^2+^-free state, suggesting an important role of Cu^2+^ in β2m oligomerization. This hexamer is, however, not amyloidogenic (Halabelian et al., 2015).

A recent study that combined NMR and MD simulations reported that the formation of hexamers by the association of dimers is an essential step in the aggregation pathway of the ΔN6 variant (Karamanos et al., 2019). The inter-dimer interfaces comprise the ABED β-sheet and the BC, DE, and FG loops, while the intra-dimer interfaces is formed by the N-terminal A strand, and the BC, DE, and FG loops. The formation of hexamers by ΔN6 increases the dynamics of the C-terminal G strand that could be responsible for initiating the formation of the cross-β structure typical of amyloid fibrils.

Table 3 provides a summary of the structural elements and residues that have been found in the interfaces of oligomeric conformations populated by β2m.

**TABLE 3:** Interfacial regions and residues found in the interfaces of β2m oligomers.

Type of oligomer	Structural elements/Interfacial residues/
wt dimer (Benyamini et al., 2003, 2005)	B strand, Phe30 and **His31** (**BC loop**), **D strand**, and **Trp60** (**DE loop**)
wt dimer (Fogolari et al., 2007)	Ile1, **Arg3** (N-terminus), **Phe56** (**D strand**), Lys58, Asp59 and **Trp60** (**DE loop**), and Arg97, Asp98, Met99 (C-terminus)
P32 dimer (Eakin et al., 2006)	Phe30 (**BC loop**), **Leu54**, **Phe56** (**D strand**) and Phe62, Tyr63 (**E strand**)
wt dimer (Mendoza et al., 2010)	Arg12 (A-strand) and Lys19 (AB loop), and Asp59 (**DE-loop**) and Tyr63 (**E strand**)
DCIM20 dimer (Colombo et al., 2012)	Arg12 (A strand), AB loop, Phe22 (B strand), Glu47 (CD loop), Ser52, Asp53 and **Leu54** (**D strand**), Tyr67, Thr68 and Glu69 (**E strand**), Phe70 and Thr71 (EF loop)
DCIM50 dimer (Colombo et al., 2012)	CD-loop, **His51** and Ser52 (**D strand**), Tyr67, Thr68 and Glu69 (**E strand**)
DCIM33 dimer (Halabelian et al., 2015)	**BC-loop**, **D strand**, **DE loop**, and Phe62, Leu64 and **Tyr66** (**E strand**)
wt dimers (Estacio et al., 2014)	**DE loop** and **BC loop Arg3** (N-terminus), **Trp60** (**DE loop**), Tyr78 (**EF loop**), Trp95 (**C-terminus**)
ΔN6 (intermediate state) dimers (Estacio et al., 2014)	Tyr10 (A strand), His13 (AB loop), Phe30 and **His 31** (**BC loop**), **Trp60** (**DE loop**) and His84 (FG loop)
wt domain-swapped dimer (Liu et al., 2011)	**D strand**, **DE loop** and **E strand**
ΔN6 domain-swapped dimer (Domanska et al., 2011)	FG loop
D76N dimer (Loureiro et al., 2019)	**Arg3** (N-terminus), Tyr10 (A strand), **Phe-56 (D strand), Trp60 (DE loop)**, Phe62 **(E strand),** Phe70 and Lys75 (EF loop), and Trp95, Arg97 (**C terminus)**
D76N dimer (Cantarutti et al., 2017; Brancolini et al., 2018)	Thr4 and Pro5 (N-terminus), Phe22 (B strand), **His31** (**BC loop**), CD loop, **D strand**, Tyr67 and Glu69 (**E strand**)
wt tetramer (Mendoza et al., 2011)	Glu50 (CD loop), **His51** and Asp53 (**D strand**), Lys91 and Lys94 (G strand) and Asp96 and Arg97 (**C-terminus**)
wt tetramer (Marcinko et al., 2019)	Glu50 (CD loop), **His51**, **Leu54** and **Phe56** (**D strand**), and Lys58 (**DE loop**)
DCIM20 tetramer (Colombo et al., 2012)	**His31** and Asp34 (**BC loop**), **His51**, **Leu54** (**D strand**), **Trp60** (**DE loop**), and the Leu64 and **Tyr66** (**E strand**)
DCIM50 tetramer (Colombo et al., 2012)	**His31** and Asp34 (**BC loop**), **His51**, **Leu54** and **Phe56** (**D strand**), **Trp60** (**DE loop**), and the Leu64 and **Tyr66** (**E strand**)
P32A tetramer (Blaho and Miranker, 2009)	**D-strand** and **DE-loop**
ΔN6 tetramer (Hall et al., 2016)	FG-loop
D76N tetramer (Loureiro et al., 2019)	**Trp60** (**DE loop**), **Arg3** (N-terminus), **Phe56** (**D strand**), Tyr 10 (A strand), and, to a lesser extent, Lys58 (**DE loop**) and Arg97 (**C-terminus**)
H13F hexamer (Calabrese et al., 2008)	Ile1 and **Arg3** (N-terminus), Tyr10 (A strand), Tyr26 (B strand), Phe30, **His31** and Asp34 (**BC loop**), **His51** and **Leu54** (**D strand**), and Tyr63 (**E strand**)
ΔN6 hexamer (Karamanos et al., 2019)	ABED β-sheet and **BC loop**, **DE loop** and FG-loop

**We highlight in bold the structural regions/residues more conserved across model systems.**

## Conclusion

We reviewed the early phase of the aggregation mechanism of protein beta-2-microglobulin (β2m), the causing agent of DRA, affecting bones and cartilages, and of a systemic amyloidosis affecting visceral organs. The focus of our analysis was the structural characterization of the monomers with the ability to trigger protein aggregation, and of the initial small oligomers formed along the amyloid pathway. Emphasis was placed on results from molecular simulations given their increasing importance as predictive tools, and as a complement to experimental studies, which allow interpreting existing data and stimulate novel experimental measurements. Simulations are particularly appropriate for the structural characterization with atomic detail of the small oligomers populating the amyloid pathway, given the possibility of isolating every conformation adopted by the protein with the desired temporal resolution.

At the monomer level, it is widely accepted that the non-native *trans* isomerization of His31-Pro32 in the intermediate I_T_ is an important—but not the only—determinant of the protein’s amyloidogenic behavior. Conformational transitions from I_T_ to other amyloid competent species must be considered to explain the protein’s ability to fibrillate. Simulation results based on native centric models predict the population of monomeric species that preserve the non-native His31-Pro32 bond, but exhibit one or both termini unstructured. While a lack of structure in one or both termini is a common feature shared by intermediate states that link the folding and aggregation landscapes of several proteins (Jarrett et al., 1993; Beland and Roucou, 2012; Neudecker et al., 2012; Zheng et al., 2015; Mompean et al., 2016; Smaoui et al., 2016; Baias et al., 2017), in the case of β2m it has only been associated with the single point mutants His31Tyr, Asp59Pro, Trp60Cys, and Asp76Asn. In simulations, an enhanced population of these intermediates typically comes in association with a decrease in thermal stability. Interestingly, results obtained with several model systems indicate that a low kinetic stability and/or low thermal stability are important physical traits that cause an enhancement of β2m’s ability to form amyloids. In line with these observations, it has been hypothesized that the specific environmental conditions occurring *in vivo* may contribute to destabilize the native structure, leading to the formation of aggregation prone monomers. In particular, the presence of collagen and glycosaminoglycans in the surface of bones and cartilages creates charge arrays that may affect the monomer’s conformational stability by destabilizing the native structure, while simultaneously increasing the nearby protein concentration (Relini et al., 2006; Esposito et al., 2012). The combination of these two effects may trigger a conformational transition of the native structure characterized by the detachment of the N- and/or C-terminal strands and by the reorganization of the aromatic side chains in the hydrophobic core (Esposito et al., 2005, 2012), which ultimately leads to the formation of a fibril-competent species capable of nucleating aggregation (Esposito et al., 2012). A scenario of this kind would explain the ability of the wt β2m to form amyloids *in vivo*. It is therefore crucial to develop models capable of reproducing the effects associated with collagen confinement and explore the conformational space of the monomer with molecular simulations.

Solving the structure of early oligomers is essential to understand the aggregation mechanism of β2m and to design therapeutic strategies that block this pathological process in the beginning. This can be accomplished through the development of drugs that bind critical interfacial residues and prevent their pathological interactions, or gene therapy, upon which these residues are substituted. The structural analysis of small oligomers highlights the importance of the DE loop, D strand and E strand (the so called DD strand interfacial region) in oligomer assemblage. The BC loop and the terminal regions also stand out as important adhesion zones. At a finer scale, Trp60 (DE loop) is one of the most prominent aggregation hotspots. The presence of His51, Leu54, Phe56, and His31 in the interfacial regions is also largely conserved across oligomers obtained from several model systems, and Phe62, Tyr63, Tyr66, Tyr67, Phe30, and Asp34 also contribute to oligomer’s stabilization, although to a lesser extent. When the N-terminus participates in self-association, interactions mediated by Arg3 are conspicuous, with Tyr10 at the N-terminal strand A being also an important player in oligomerization. Analogously, participation of the C-terminus in the oligomer’s interfacial region proceeds mainly by interactions mediated by Arg97.

While the results outlined above are certainly important one should keep in mind the existing uncertainty regarding the biological significance of the structural variants, and the fact that certain oligomers that have been analyzed experimentally are not amyloidogenic. The resilience of the wt form to aggregate *in vitro* under physiological conditions also motivates the establishment of an alternative mechanism for β2m aggregation exclusively based on the wt form in conditions that typically occur *in vivo.* Future research on this topic should therefore evolve along a direction where *in vivo* conditions are replicated both in the simulated environment of the test tube and in molecular simulations.

## Author Contributions

PF: conceptualization, visualization. RL: original draft. PF and RL: writing review and editing. All authors contributed to the article and approved the submitted version.

## Conflict of Interest

The authors declare that the research was conducted in the absence of any commercial or financial relationships that could be construed as a potential conflict of interest.
